# Mechanism of Yiqi Yangying Heluo Formula in the Treatment of IgA Nephropathy by Affecting Gd-IgA1 Based on BAFF Molecular Level and T Lymphocyte Immunity

**DOI:** 10.1155/2023/5124034

**Published:** 2023-01-10

**Authors:** Ying Liang, Qin Zeng, Xin-Hui Wang, Lei Yan, Ren-Huan Yu

**Affiliations:** Department of Nephrology, Xiyuan Hospital of China Academy of Chinese Medical Sciences, Beijing, China

## Abstract

**Background:**

Galactose-deficient IgA1 (Gd-IgA1) is a critical initiating factor in the pathogenesis of IgA nephropathy (IgAN), which plays an important role in the diagnosis and evaluation of this disease. Moreover, the whole pathogenesis process has an intimate association with the immune response of T and B lymphocytes and their inflammatory factors. There is no specific therapy for IgAN at present. Yiqi Yangyin Formula can significantly reduce urinary protein and hematuria in patients with IgAN. Yiqi Yangying Heluo Formula (YYHF) is optimized on the basis of the above prescription, but its specific mechanism remains to be further studied.

**Methods:**

The effect of YYHF on urinary protein and urinary red blood cell count in patients with IgAN was observed by a self-controlled clinical study before and after treatment. On this basis, flow cytometry was used to detect the proportion of T lymphocyte subsets in peripheral blood of patients with IgAN before and after treatment and healthy controls. Meanwhile, the levels of Gd-IgA1, B cell activation factor (BAFF), and their cytokines (IL-4, IL-6, and IL-17) in peripheral blood were detected by enzyme-linked immunosorbent assay. The changes in mechanism-related indicators of the two groups were observed and subject to correlation analysis.

**Results:**

(1) Compared with the levels before treatment, 24-hour urinary protein content decreased by 47.7% and urinary red blood cell number decreased by 67% in patients with IgAN intervened by YYHF after 48 weeks of follow-up. (2) Compared with the healthy control group, patients with IgAN showed a significantly increased proportion of Th1 cells, Th17 cells, Th1/Th2, Th1/Treg, Th2/Treg, and Th17/Treg, obviously reduced proportion of Th2 cells and Treg cells, and evidently elevated levels of Gd-IgA1, BAFF, and their cytokines (IL-4, IL-6, and IL-17) in the peripheral blood. (3) Following 48 weeks of follow-up after intervention treatment with YYHF, the levels of Gd-IgA1, BAFF, IL-6, and IL-17 were significantly lower, but the level of IL-4 was higher in peripheral blood of patients with IgAN than those before treatment and after 24 weeks of treatment; simultaneously, the proportion of Th1 cells, Th17 cells, Th1/Th2, Th1/Treg, Th2/Treg, and Th17/Treg decreased while that of Th2 cells and Treg cells increased after 48 weeks of follow-up compared with that before treatment in peripheral blood of patients with IgAN. (4) The results of correlation analysis revealed that the level of Gd-IgA1 in peripheral blood of patients with IgAN was positively correlated with the level of BAFF, as well as the proportion of Th1 cells, Th17 cells, Th1/Th2, IL-6, and IL-17 levels, and negatively correlated with the proportion of Treg cells. In addition, the level of Gd-IgA1 in peripheral blood was positively correlated with proteinuria, yet without correlation with hematuria.

**Conclusion:**

YYHF can reduce the quantitative level of 24 h urinary protein and urinary red blood cell count in patients with IgAN. Patients with IgAN have obvious T cell immune imbalance. YYHF can significantly reduce the level of Gd-IgA1 in patients with IgAN, and its mechanism may be explained by the reduced level of BAFF in peripheral blood and improved immune balance of T cells.

## 1. Background

IgA nephropathy (IgAN) is the most common primary glomerular disease. Galactose-deficient IgA1 (Gd-IgA1), produced by the mucosal immune system, is a critical initiating factor in the pathogenesis of IgAN [1]. Multiple studies have documented that Gd-IgA1 is crucial in the diagnosis and evaluation of IgAN; and the increase of circulating Gd-IgA1 is significantly positively correlated with the increased risk of proteinuria and deterioration of renal function in patients with IgAN [2–4].

The pathogenicity of Gd-IgA1 consists of T cell-dependent and non-T cell-dependent pathways. T lymphocytes play an important role in abnormal IgA1 galactosylation. Meanwhile, the non-T cell-dependent pathway is mediated by a proliferation-inducing ligand (APRIL) and B cell activation factor (BAFF). This process is related to the IgA class switching during the activation of B cells into plasma cells. Both APRIL and BAFF play an extremely important role in B cell differentiation, immunoglobulin class switching, maintaining B cell survival, and inhibiting apoptosis. It has been previously confirmed that transgenic mice overexpressing BAFF showed increased serum IgA levels and increased deposition of IgA immune complexes in the renal glomerular mesangial region, suggesting that BAFF may be involved in the progression of IgAN [5]. In addition, the concentration of serum BAFF in patients with IgAN was reported to be higher than that in patients without IgAN, and it was associated with the clinicopathological characteristics of the former patients.

According to previous research [6], Yiqi Yangyin Formula can significantly reduce urinary protein and hematuria in patients with IgAN. Yiqi Yangying Heluo Formula (YYHF) is optimized on the basis of the above prescription, and it is an empirical traditional Chinese medicine prescription for clinical IgAN treatment in China, but its specific mechanism remains to be further studied. Through self-controlled clinical study before and after treatment, it was designed to observe the effect of the YYHF on clinical indicators of patients with IgAN and confirm the effectiveness of this recipe in the treatment of IgAN. Furthermore, on the basis of the verified effectiveness, the relevant factors affecting the level of Gd-IgA1 were analyzed through the intervention of BAFF level in peripheral blood, the proportion of T lymphocyte subsets, and the changes in their cytokine levels in patients with IgAN, so as to explore the pathogenesis of IgAN intervened by YYHF. The report is as follows.

## 2. Subjects and Methods

### 2.1. Subjects of the Study

The IgAN group consisted of 25 patients with IgAN diagnosed by renal biopsy in the Outpatient and Inpatient Department of Nephrology, Xiyuan Hospital, China Academy of Chinese Medical Sciences, from December 2018 to December 2020. In the same period, ten matched healthy volunteers in the Physical Examination Center of Xiyuan Hospital were selected as the healthy control group (HC group).

### 2.2. Inclusion and Exclusion Criteria

#### 2.2.1. Inclusion Criteria of the IgAN Patients

The inclusion criteria of the IgAN patients are as follows: (1) diagnosed as IgAN by renal puncture biopsy; (2) 24 h urinary protein quantification of 0.5-3 g/d and eGFR > 60 mL/min/1.73 m^2^; (3) age ranging 18-60 years; (4) conforming to qi-yin deficiency syndrome of traditional Chinese medicine; and (5) agreeing with and signing the informed consent.

#### 2.2.2. Exclusion Criteria of the IgAN Patients

The exclusion criteria of the IgAN patients are as follows: (1) complicated with major systemic diseases of the cardiocerebrovascular system, nervous system, and immune system; (2) use of glucocorticoids, immunosuppressants, and antibiotics in recent 3 months; (3) secondary nephrosis such as systemic lupus erythematosus nephritis, hepatitis B-related nephritis, anaphylactoid purpura nephritis, and diabetic nephropathy; (4) pregnant or preparing for pregnancy and lactating woman; (5) participated in clinical trials of other drugs within the last month; (6) infections such as respiratory tract infection and urinary system infection within 1 month; and (7) alcoholics, drug abusers, and addicts.

#### 2.2.3. Inclusion Criteria of the HC Group

The inclusion criteria of the HC group are as follows: healthy adults close to patients with IgAN in age, gender, family genetic history, and BMI; undergoing strict clinical physical examination and screening, with normal blood pressure (systolic blood pressure < 130 mmHg and diastolic blood pressure < 80 mmHg); within the normal ranges in the electrocardiogram, chest X-ray, abdominal ultrasound, blood biochemistry, routine blood test, routine urine test, and routine stool test; and no history of hypertension, coronary heart disease, diabetes, gastrointestinal diseases, or gastrointestinal surgery.

### 2.3. Protocols of Study

#### 2.3.1. Experimental Medication

The basic YYHF was applied for the experimental medication of the enrolled patients, with modifications according to the symptoms. The recipe was administered orally, one dose per day for two times. The course of treatment was 24 weeks, and patients were followed up for 48 weeks. YYHF is made of 5 different Chinese herbs, including Radix Astragali (Hangqi), Radix Stephania tetrandra (Fangji), Rhizoma polygonati (Huangjing), Folium nelumbinis (Heye), and Perillae Folium (Zisuye).

#### 2.3.2. Basic Treatment

RAS blockers (ACEI or ARB) were used to control blood pressure, and the management of blood pressure was referred to the KDIGO guidelines in 2012.

### 2.4. Outcome Measures

#### 2.4.1. Routine Laboratory Tests

Routine laboratory tests included routine blood tests, liver function, renal function, plasma albumin, blood IgA, blood C3, and blood lipids; 24 h urinary protein quantification and urinary red blood cell count; and estimated glomerular filtration rate calculated based on CKD-EPI formula.

#### 2.4.2. Mechanism-Related Indicators

Relevant indicators consisted of T lymphocyte subsets (Th1, Th2, Treg, and Th17), peripheral blood Gd-IgA1, BAFF, and cytokine levels (IL-4, IL-6, and IL-17).

### 2.5. Experimental Methods

Fasting venous blood samples were obtained from patients with IgAN before and after treatment and healthy controls using BD Vacutainer® CPT™ Cell Preparation Tubes. The upper serum was stored at -80°C for further use, and the peripheral blood mononuclear cells (PBMC) at the bottom of the tub were collected immediately for flow cytometry.

#### 2.5.1. Detection of the Proportion of Th1, Th2, Th17, and Treg Cells in Peripheral Blood by Flow Cytometry

An appropriate amount of different fluorescently labeled antibodies (all selected fluorescent antibodies were obtained from BD Biosciences) was added to the prepared PBMC cell suspension. Specifically, PerCP-CyTM5.5 mouse anti-human CD, APC-H7 mouse anti-human CD4, PE mouse anti-human CD183 (CXCR3), and PE-Cy7 mouse anti-human CD196 (CCR6) were added into the tube containing Th1/2/17, while PerCP-CyTM5.5 mouse anti-human CD3, APC-H7 mouse anti-human CD4, Alexa Fluor® 647 mouse anti-human CD127, and BB515 mouse anti-human CD25 were added into the tube containing Treg. After the incubation in the dark, the cells were cleaned and resuspended for flow cytometry using FACSCalibur flow cytometer (BD Biosciences, USA). The results were analyzed by CellQuest software.

#### 2.5.2. Detection of the Levels of Gd-IgA1, BAFF, and Cytokines (IL-4, IL-6, and IL-17) in Peripheral Blood by ELISA

The levels of Gd-IgA1, BAFF, and cytokines (IL-4, IL-6, and IL-17) in peripheral blood were detected by enzyme-linked immunosorbent assay (ELISA) using corresponding ELISA kits (R&D, USA). The OD value was detected by Microplate Reader (Bio-Rad, USA), and the standard curve was generated by regression fitting with MS Office Excel.

### 2.6. Statistical Analysis

All data of this study were analyzed by SPSS21.0 software. The measurement data were expressed as mean ± standard deviation, and the counting data were presented by incidence or constituent ratio. A Fisher's precision probability test was used to compare the rates or constituent ratios between groups. All the data were tested for normality and homogeneity of variance. The two groups of data with normal distribution were compared by independent sample *t*-test, and those without normal distribution were compared by Mann-Whitney *U*-test. Paired sample *t*-test was used for data with normal distribution before and after treatment, and paired rank sum test was used for data without normal distribution. The Spearman correlation analysis was used to analyze the correlation between the two variables. *P* < 0.05 meant that the difference was statistically significant.

## 3. Results

### 3.1. Comparison of General Data

A total of 25 patients with IgAN confirmed by renal biopsy were included in this study, including 15 males and 10 females, with an average age of 41.70 ± 10.46 years (24-58 years), median age of 43 years, and median duration of 3 years (1-10 years). Meanwhile, there were 10 subjects in the HC group, including 6 males and 4 females, with an average age of 42.89 ± 11.52 years (21-58 years). Sixty-eight percent of the 25 patients with IgAN had hypertension, and their blood pressure was all controlled at <130/80 mmHg. There was no significant difference in gender ratio, age, and BMI between the two groups (*P* > 0.05) (Table 1).

### 3.2. Effect of YYHF on Main Clinical Indicators in Patients with IgAN

According to the detection results of clinical indicators in 25 IgAN patients intervened by YYHF, compared with the levels before treatment, the 24 h urinary protein quantitative level and IgA/C3 level of IgA patients decreased after 24 weeks of treatment and follow-up to 48 weeks, both of which were lower after follow-up to 48 weeks than those after 24 weeks of treatment (all *P* < 0.05). Meanwhile, urinary red blood cell count was observed to be decreased in patients with IgAN who were followed up to 48 weeks compared with that before treatment. In addition, there was no significant difference in other indicators before and after treatment (*P* > 0.05) (Table 2).

### 3.3. Effect of YYHF on the Levels of Gd-IgA1 and BAFF in Peripheral Blood of Patients with IgAN

**Figure 1:**
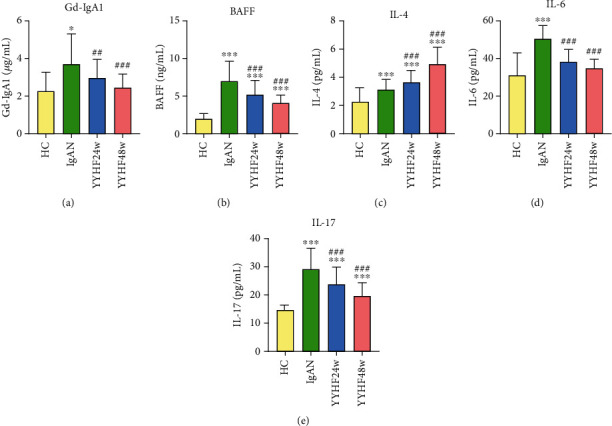
Effect of Yiqi Yangying Heluo Formula on the levels of Gd-IgA1, BAFF, IL-4, IL-6, and IL-17 in peripheral blood of patients with IgAN. Note: ^∗^Compared with the HC group, *P* < 0.05. ^∗∗∗^Compared with the HC group, *P* < 0.001. ^##^Compared with the level before treatment, *P* < 0.01. ^###^Compared with the level before treatment, *P* < 0.001.

Based on the detection results of Gd-IgA1 and BAFF levels in 25 IgAN patients intervened by YYHF, the levels of Gd-IgA1 and BAFF in peripheral blood of patients with IgAN were significantly higher than those in the HC group. After 24 weeks of treatment, the levels of Gd-IgA1 and BAFF in peripheral blood decreased compared with those before treatment, with a significant decrease after 48 weeks of follow-up (all *P* < 0.05) (Table 3 and Figures 1(a) and 1(b)).

### 3.4. Effect of YYHF on the Levels of IL-4, IL6, and IL-17 in Peripheral Blood of Patients with IgAN

The detection results of IL-4, IL-6, and IL-17 levels in 25 IgAN patients intervened by YYHF are shown in Table 4 and Figures 1(c)–1(e). After 24 weeks of treatment and 48 weeks of follow-up with YYHF, the levels of IL-6 and IL-17 in peripheral blood of patients with IgAN were significantly lower than those before treatment; besides, the level of IL-4 in peripheral blood of these patients was higher than those before treatment (all *P* < 0.05).

### 3.5. Effect of YYHF on Th1/Th2/Th17/Treg Cell Levels in Peripheral Blood of Patients with IgAN

**Figure 2:**
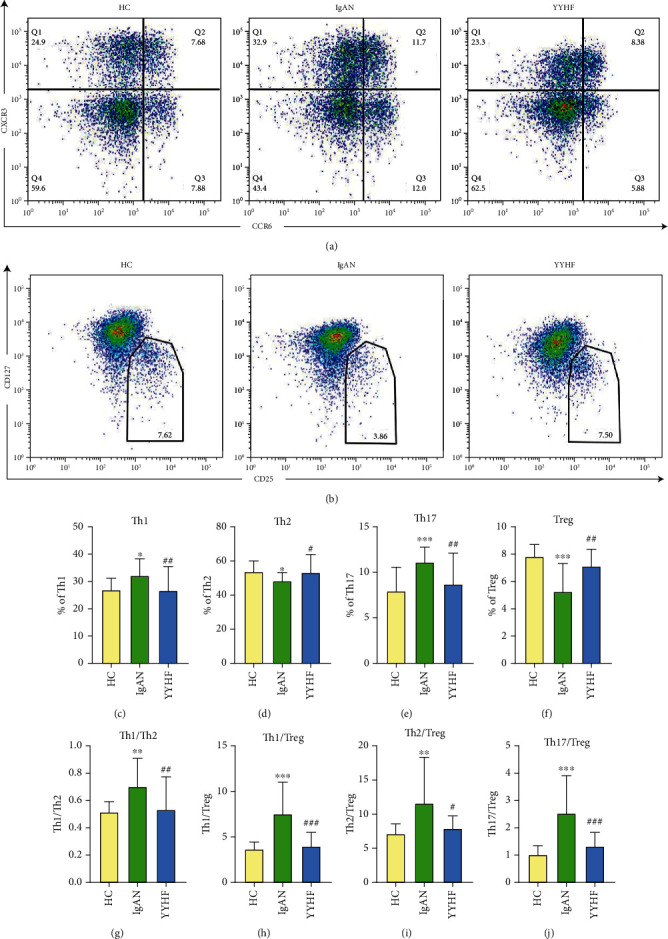
Effect of Yiqi Yangying Heluo Formula on Th1/Th2/Th17/Treg cell level in peripheral blood of patients with IgAN and flow cytometry diagrams. Note: ^∗^Compared with the HC group, *P* < 0.05. ^∗∗^Compared with the HC group, *P* < 0.01. ^∗∗∗^Compared with the HC group, *P* < 0.001. ^#^Compared with the level before treatment, *P* < 0.05. ^##^Compared with the level before treatment, *P* < 0.01. ^###^Compared with the level before treatment, *P* < 0.001.

Flow cytometry was performed to measure the percentages of Th1/Th2/Th17/Treg cell subsets from peripheral blood (Figures 2(a) and 2(b)).  Table 5 and Figures 2(c)–2(j) show the analysis results of Th1/Th2/Th17/Treg cell subsets in 25 IgAN patients intervened by YYHF. Compared with the HC group, there existed an increasing trend in the proportion of Th1 cells, Th17 cells, Th1/Th2, Th1/Treg, Th2/Treg, and Th17/Treg in peripheral blood of patients with IgAN before treatment, while the proportion of Th2 cells and Treg cells decreased. Following 48 weeks of follow-up after intervention treatment with YYHF, the proportion of Th1 cells, Th17 cells, Th1/Th2, Th1/Treg, Th2/Treg, and Th17/Treg decreased while that of Th2 cells and Treg cells increased compared with that before treatment in peripheral blood of patients with IgAN (all *P* < 0.05).

### 3.6. Correlation Analysis of Gd-IgA1 Level in Peripheral Blood with BAFF, T Lymphocyte Subsets, and Their Cytokines in Patients with IgAN

**Figure 3:**
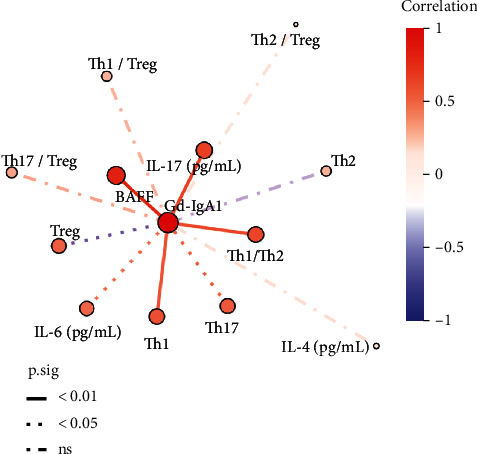
Correlation analysis of Gd-IgA1 level in peripheral blood with BAFF and T lymphocyte immunity in patients with IgAN.

The results of correlation analysis between Gd-IgA1 and BAFF revealed that the level of Gd-IgA1 in peripheral blood of patients with IgAN was positively correlated with the level of BAFF, as well as the proportion of Th1 cells, Th17 cells, Th1/Th2, IL-6, and IL-17 levels, and negatively correlated with the proportion of Treg cells (all *P* < 0.05, Figure 3).

### 3.7. Correlation Analysis of Gd-IgA1 Level in Peripheral Blood with Proteinuria and Hematuria in Patients with IgAN

**Figure 4:**
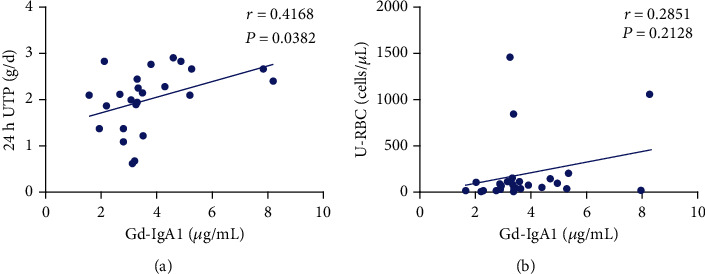
Correlation analysis of Gd-IgA1 level in peripheral blood with proteinuria and hematuria in patients with IgAN.

Correlation analysis results of Gd-IgA1 with proteinuria and hematuria are shown in Figures 4(a) and 4(b). The level of Gd-IgA1 in peripheral blood was positively correlated with proteinuria (*P* < 0.05), yet without correlation with hematuria (*P* > 0.05).

## 4. Discussion

IgAN is the most common primary glomerulonephritis in the world, especially in young people aged 20 to 40 years [7]. It accounts for 45%-50% of primary glomerulonephritis in China, and about 15%-40% of patients will progressively develop the end-stage renal disease within 20 years after diagnosis [8].

Our research group proposed that YYHF is an effective and safe therapeutic option for IgAN. This prescription can significantly reduce urinary protein and hematuria in patients with IgAN, with definite clinical curative effects [6, 9]. YYHF is optimized on the basis of Yiqi Yangyin Formula. This study included 25 patients with IgAN for analysis. According to the results of the effect of YYHF on clinical indicators after the intervention, compared with the levels before treatment, there were decreased trends in 24 h urinary protein quantitative level and IgA/C3 level of IgAN patients after 24 weeks of treatment and follow-up to 48 weeks, both of which were lower after follow-up to 48 weeks than those after 24 weeks of treatment. Moreover, urinary red blood cell count in patients with IgAN who were followed up to 48 weeks decreased compared with that before treatment. Our study emphasized the exploration of its mechanism.

In recent decades, great progress has been made in clarifying the pathogenesis of IgAN. It is currently believed that there are significant glycosylation abnormalities in circulating IgA1 in patients with IgAN, mainly galactose deficiency, and the IgA deposited in the kidney is also mainly Gd-IgA1. Elevated circulating Gd-IgA1 is currently considered to be a key initiating molecule in the pathogenesis of IgAN. Various clinical and basic studies recently have suggested that Gd-IgA1 plays an important role in the diagnosis and evaluation of IgAN; and there is a significant positive correlation between the increase of circulating Gd-IgA1 and the increased risk of severe proteinuria and deterioration of renal function in patients with IgAN [2–4]. The level of Gd-IgA1 is related to the severity and prognosis of the disease and can be affected by treatment. For instance, Kim et al. [10] found in their research that the use of immunosuppressants in patients with IgAN after renal transplantation could influence the serum level of Gd-IgA1 to some extent. In their study, in 64 IgAN patients who underwent renal transplantation, there was a significant decrease in the level of Gd-IgA1 within 0-3 months of immunosuppressant use, suggesting that the use of glucocorticoids may have an impact on the level of Gd-IgA1. Meanwhile, Kosztyu et al. reported recently that the use of glucocorticoids can reduce the level of Gd-IgA1 in patients with IgAN [11]. In our study, the level of Gd-IgA1 in peripheral blood of patients with IgAN was significantly higher than that of healthy controls. After 24 weeks of treatment with YYHF, the level of Gd-IgA1 in peripheral blood reduced compared with that before treatment, accompanied by a significant decrease of Gd-IgA1 and lower quantitative level of urinary protein at 48 weeks of follow-up, which were consistent with the findings of previous research. In addition, further correlation analysis revealed that the level of Gd-IgA1 in peripheral blood was positively correlated with proteinuria, yet without correlation with hematuria.

According to previous research on the pathogenesis of this disease, patients with IgAN have an imbalance of T cell subsets and their cytokines; and it was observed that the percentage of effector memory Th1 cells was relatively higher in peripheral blood of patients with IgAN [12]. Meanwhile, increased expression of Th1 transcription factor T-bet and decreased expression of Th2 transcription factor GATA3 were detected in the urine of patients with IgAN [13]. Besides, IgAN patients with poor prognoses have been confirmed to have a higher percentage of effector memory Th17 cells. With respect to the above, these T lymphocyte subsets may be associated with disease activity and related to mesangial cell activation (deposition of Gd-IgA1 and related immune complexes), which may further induce the secretion of two cytokines (IL-6 or TGF-*β*) that are related to the polarization of Th17 [12]. Th17 cells are defined as a new subset of CD4^+^ T-helper cells found in recent years, with intimate association with Treg cells in differentiation regulation. Under physiological conditions, Th17 and Treg cells are in dynamic balance, and there may be an imbalance of Th17/Treg cells in the case of dysfunction of the organism. The imbalance of T lymphocyte subsets and their interleukins is related to the clinical characteristics of IgAN, including the occurrence and severity of proteinuria, the increase of serum creatinine, and the decrease of GFR and hematuria. As evidenced by prior studies, the severity of 24-hour proteinuria was positively correlated with serum IL-21 and IL-17; the decrease of Treg cell level could cause the increase of proteinuria in patients with IgAN; and eGFR level was positively correlated with the proportion of activated Treg cells and negatively correlated with the proportion of Tfh cells. Consistent with the research results of Sun et al. [14], further research on B cells and IgA1-secreting cells in patients with IgAN showed that IL-4 was an important influential factor. Furthermore, another study revealed that the proinflammatory Th1 and Th17 responses might be involved in the mechanism of IgAN and stimulate anti-inflammatory Th2 and Treg cells and then feedback to downregulate the proinflammatory response during the pathogenesis of IgAN. Therefore, in their study, compared with the HC group, the levels of serum IL-2, IL-4, IL-10, IL-17A, and IFN-*γ* were significantly increased in patients with IgAN; and there would be an obvious increase in the levels of serum IL-4 and IL-10 after treatment [15]. In the present study, before treatment, patients with IgAN increased were observed with an increased proportion of Th1 cells, Th17 cells, Th1/Th2, Th1/Treg, Th2/Treg, and Th17/Treg in peripheral blood, while decreased proportion of Th2 cells and Treg cells. After intervention with YYHF, patients with IgAN showed a decreased proportion of Th1 cells, Th17 cells, Th1/Th2, Th1/Treg, Th2/Treg, and Th17/Treg in peripheral blood, while an increased proportion of Th2 cells and Treg cells at 48 weeks of follow-up compared with those before treatment. Simultaneously, in terms of the effect on the levels of cytokines, the levels of IL-4, IL-6, and IL-17 in patients with IgAN were increased than those of the HC group before treatment, while after 24 weeks of treatment using YYHF and 48 weeks of follow-up, the levels of IL-6 and IL-17 were significantly lower and that of IL-4 was increased than those before treatment in peripheral blood of patients with IgAN. The aforementioned results in our study are consistent with many studies at home and abroad.

BAFF, also known as a B lymphocyte stimulator, can bind to B cells to induce their proliferation and maturation, prolong survival, promote differentiation, and regulate peripheral B cell homeostasis. As reported by McCarthy et al. [5], mice overexpressing BAFF showed significantly increased circulating levels of aberrantly glycosylated IgA, increased mesangial IgA deposition, and higher incidences of hematuria and proteinuria. BAFF transgenic mice showed elevated serum IgA levels and deposition of IgA immune complexes in the renal glomerular mesangial region, suggesting that BAFF may be involved in the progression of IgAN. In our study, the level of BAFF in peripheral blood of patients with IgAN was significantly higher than that of the HC group. After 24 weeks of treatment, the level of BAFF decreased compared with that before treatment, with a further decrease significantly at 48 weeks of follow-up. Simultaneously, correlation analysis suggested that peripheral blood Gd-IgA1 was positively correlated with the level of BAFF, as well as the proportion of Th1 cells, Th17 cells, Th1/Th2, IL-6, and IL-17, and negatively correlated with the proportion of Treg cells. Similarly, Yanagawa et al. also believed that BAFF might be positively correlated with the severity of IgAN through the T cell-independent mode of IgA class switching, and exhibited an important correlation with the increase of Gd-IgA1 level, suggesting a novel mechanism leading to the pathogenesis of IgAN.

## 5. Conclusion

1. YYHF can reduce the quantitative level of 24 h urinary protein and urinary red blood cell count in patients with IgAN
2. Patients with IgAN have T/B cell immune imbalance, which is mainly manifested in the increased level of BAFF in peripheral blood, as well as the imbalanced proportion of T cell subsets and the levels of their cytokines
3. Patients with IgAN have a significantly elevated level of Gd-IgA1 than that in healthy controls. Besides, the level is significantly correlated with the level of BAFF, the proportion of T cell subsets, and the levels of their cytokines
4. YYHF can significantly reduce the level of Gd-IgA1 in patients with IgAN, and its mechanism may be explained by the reduced level of BAFF in peripheral blood and improved immune balance of T cells

## Figures and Tables

**Table 1:** Comparison of general data.

Items	IgAN group (*n* = 25)	HC group (*n* = 10)	*P*
Gender (male/female)	15/10	6/4	0.313
Age (years) (mean, SD) (range)	41.70 ± 10.46 (24-58)	42.89 ± 11.52 (21-58)	0.426
BMI (kg/m^2^) (mean, SD)	24.19 ± 2.17	24.97 ± 1.98	0.582
Hypertension (%)	68%	/	/

Note: *P* value, the result of comparison between groups. BMI: body mass index.

**Table 2:** Effect of Yiqi Yangying Heluo Formula on main clinical indicators in patients with IgAN ($\bar{x}\pm \text{s}$).

Items	Before treatment	12 weeks of treatment	24 weeks of treatment	48 weeks of follow-up
24 h UTP (g/d) (mean, SD)	1.99 ± 0.66	1.71 ± 0.57^∗^	1.47 ± 0.54^∗^^#^	1.04 ± 0.42^∗^^#▲^
U-RBC (cells/*μ*L) (mean, SD)	196.57 ± 360.16	128.88 ± 236.46^∗^	92.82 ± 167.18^∗^^#^	64.92 ± 85.42^∗^^#^
ALB (g/L) (mean, SD)	41.41 ± 3.43	40.21 ± 3.72	40.49 ± 3.25	40.69 ± 3.08
IgA/C3 (mean, SD)	3.12 ± 0.48	2.78 ± 0.40^∗^	2.51 ± 0.32^∗^^#^	2.11 ± 0.27^∗^^#▲^
Scr (*μ*mol/L) (mean, SD)	68.24 ± 17.18	67.84 ± 16.49	68.76 ± 16.52	68.80 ± 13.62
eGFR (mL/min∗1.73 m^2^) (mean, SD)	100.91 ± 20.32	101.00 ± 19.80	100.78 ± 21.00	103.21 ± 19.00
BUN (mmol/L) (mean, SD)	12.43 ± 3.11	14.28 ± 4.32	14.10 ± 3.34	13.93 ± 4.09
TC (mmol/L) (mean, SD)	4.99 ± 1.21	5.07 ± 0.86	4.84 ± 0.69	4.91 ± 1.04
TG (mmol/L) (mean, SD)	2.63 ± 1.32	2.56 ± 1.52	2.44 ± 1.37	2.34 ± 1.23
ALT (U/L) (mean, SD)	18.28 ± 8.58	14.36 ± 5.14	16.55 ± 5.18	15.81 ± 5.26
AST (U/L) (mean, SD)	19.52 ± 7.72	18.22 ± 6.43	18.24 ± 4.91	18.03 ± 3.95
RBC (10^12^/L) (mean, SD)	4.46 ± 0.57	4.46 ± 0.64	4.35 ± 0.74	4.38 ± 0.68
HB (g/L) (mean, SD)	138.80 ± 17.81	135.24 ± 19.30	134.80 ± 17.65	133.88 ± 17.49
PLT (10^9^/L) (mean, SD)	284.52 ± 66.49	280.48 ± 57.22	275.36 ± 60.46	276.72 ± 63.29
WBC (10^9^/L) (mean, SD)	6.33 ± 1.86	6.56 ± 2016	6.26 ± 2.00	6.31 ± 1.88

Note: ^∗^Compared with the level before treatment, *P* < 0.05. ^#^Compared with the level at 12 weeks of treatment, *P* < 0.05. ^▲^Compared with the level at 24 weeks of treatment, *P* < 0.05. 24 h UTP: 24-hour urinary protein; U-RBC: urinary red blood cell; ALB: serum albumin; Scr: serum creatinine; eGFR: estimated glomerular filtration rate; BUN: blood urea nitrogen; TC: serum total cholesterol; TG: serum triglyceride; ALT: serum alanine aminotransferase; AST: serum aspartate aminotransferase; RBC: red blood cell; HB: hemoglobin; PLT: platelet; WBC: white blood cell.

**Table 3:** Effect of Yiqi Yangying Heluo Formula on the levels of Gd-IgA1 and BAFF in peripheral blood of patients with IgAN ($\bar{x}\pm \text{s}$).

Items	HC group	IgAN group		
		Before treatment	24 weeks of treatment	48 weeks of follow-up
Gd-IgA1 (*μ*g/mL)	2.28 ± 1.00	3.71 ± 1.60^∗^	2.98 ± 0.99^#^	2.47 ± 0.71^#▲^
BAFF (ng/mL)	1.96 ± 0.67	6.98 ± 2.70^∗^	5.22 ± 1.82^∗^^#^	4.16 ± 1.02^∗^^#▲^

Note: ^∗^Compared with the HC group, *P* < 0.05. ^#^Compared with the level before treatment, *P* < 0.05. ^▲^Compared with level at 24 weeks of treatment, *P* < 0.05.

**Table 4:** Effect of Yiqi Yangying Heluo Formula on the levels of IL-4, IL6, and IL-17 in peripheral blood of patients with IgAN ($\bar{x}\pm \text{s}$).

Items	HC group	IgAN group		
		Before treatment	24 weeks of treatment	48 weeks of follow-up
IL-4 (pg/mL)	2.23 ± 0.99	3.09 ± 0.70^∗^	3.58 ± 0.85^∗^^#^	4.85 ± 1.21^∗^^#▲^
IL-6 (pg/mL)	30.89 ± 11.72	50.08 ± 6.84^∗^	38.32 ± 6.10^#^	34.52 ± 4.74^#▲^
IL-17 (pg/mL)	14.66 ± 1.65	28.88 ± 7.37^∗^	23.58 ± 6.40^∗^^#^	19.39 ± 4.77^∗^^#▲^

Note: ^∗^Compared with the HC group, *P* < 0.05. ^#^Compared with the level before treatment, *P* < 0.05. ^▲^Compared with level at 24 weeks of treatment, *P* < 0.05.

**Table 5:** Effect of Yiqi Yangying Heluo Formula on Th1/Th2/Th17/Treg cell levels in peripheral blood of patients with IgAN ($\bar{x}\pm \text{s}$).

Items	HC group	IgAN group	
		Before treatment	48 weeks of follow-up
Th1 (%)	26.89 ± 4.29	32.30 ± 6.25^∗^	26.45 ± 9.17^#^
Th2 (%)	51.15 ± 9.24	49.35 ± 7.13	53.20 ± 10.44
Th17 (%)	7.82 ± 2.67	11.01 ± 1.76^∗^	8.62 ± 3.39^#^
Treg (%)	7.79 ± 0.91	5.22 ± 2.11^∗^	7.13 ± 1.24^#^
Th1/Th2	0.54 ± 0.11	0.68 ± 0.22^∗^	0.53 ± 0.25^#^
Th1/Treg	3.52 ± 0.84	7.36 ± 3.55^∗^	3.84 ± 1.65^#^
Th2/Treg	6.68 ± 1.67	12.00 ± 8.33^∗^	7.69 ± 2.01^#^
Th17/Treg	1.02 ± 0.35	2.52 ± 0.38^∗^	1.28 ± 0.56^#^

Note: ^∗^Compared with the HC group, *P* < 0.05. ^#^Compared with the level before treatment, *P* < 0.05.

## Data Availability

All data relevant to the study are included in the article; further enquiries can be directed to the corresponding author.
